# A Systematic Review on the Pharmacokinetics of Cannabidiol in Humans

**DOI:** 10.3389/fphar.2018.01365

**Published:** 2018-11-26

**Authors:** Sophie A. Millar, Nicole L. Stone, Andrew S. Yates, Saoirse E. O'Sullivan

**Affiliations:** ^1^Division of Medical Sciences and Graduate Entry Medicine, School of Medicine, University of Nottingham, Royal Derby Hospital, Derby, United Kingdom; ^2^Artelo Biosciences, San Diego, CA, United States

**Keywords:** pharmacokinetics, endocannabinoid system, bioavailability, CMAX, TMAX, half life, plasma clearance, volume of distribution

## Abstract

**Background:** Cannabidiol is being pursued as a therapeutic treatment for multiple conditions, usually by oral delivery. Animal studies suggest oral bioavailability is low, but literature in humans is not sufficient. The aim of this review was to collate published data in this area.

**Methods:** A systematic search of PubMed and EMBASE (including MEDLINE) was conducted to retrieve all articles reporting pharmacokinetic data of CBD in humans.

**Results:** Of 792 articles retireved, 24 included pharmacokinetic parameters in humans. The half-life of cannabidiol was reported between 1.4 and 10.9 h after oromucosal spray, 2–5 days after chronic oral administration, 24 h after i.v., and 31 h after smoking. Bioavailability following smoking was 31% however no other studies attempted to report the absolute bioavailability of CBD following other routes in humans, despite i.v formulations being available. The area-under-the-curve and C_max_ increase in dose-dependent manners and are reached quicker following smoking/inhalation compared to oral/oromucosal routes. C_max_ is increased during fed states and in lipid formulations. T_max_ is reached between 0 and 4 h.

**Conclusions:** This review highlights the paucity in data and some discrepancy in the pharmacokinetics of cannabidiol, despite its widespread use in humans. Analysis and understanding of properties such as bioavailability and half-life is critical to future therapeutic success, and robust data from a variety of formulations is required.

## Introduction

The *Cannabis sativa* plant contains more than a hundred phytocannabinoid compounds, including the non-psychotomimetic compound cannabidiol (CBD) (Izzo et al., 2009). CBD has attracted significant interest due to its anti-inflammatory, anti-oxidative and anti-necrotic protective effects, as well as displaying a favorable safety and tolerability profile in humans (Bergamaschi et al., 2011), making it a promising candidate in many therapeutic avenues including epilepsy, Alzheimer's disease, Parkinson's disease, and multiple sclerosis. GW pharmaceuticals have developed an oral solution of pure CBD (Epidiolex®) for the treatment of severe, orphan, early-onset, treatment-resistant epilepsy syndromes, showing significant reductions in seizure frequency compared to placebo in several trials (Devinsky et al., 2017, 2018a; Thiele et al., 2018). Epidiolex® has recently received US Food and Drug Administration (FDA) approval (GW Pharmaceuticals, 2018). CBD is also being pursued in clinical trials in Parkinson's disease, Crohn's disease, society anxiety disorder, and schizophrenia (Crippa et al., 2011; Leweke et al., 2012; Chagas et al., 2014; Naftali et al., 2017), showing promise in these areas. Additionally, CBD is widely used as a popular food supplement in a variety of formats for a range of complaints. It is estimated that the CBD market will grow to $2.1 billion in the US market in consumer sales by 2020 (Hemp Business, 2017).

From previous investigations including animal studies, the oral bioavailability of CBD has been shown to be very low (13–19%) (Mechoulam et al., 2002). It undergoes extensive first pass metabolism and its metabolites are mostly excreted via the kidneys (Huestis, 2007). Plasma and brain concentrations are dose-dependent in animals, and bioavailability is increased with various lipid formulations (Zgair et al., 2016). However, despite the breadth of use of CBD in humans, there is little data on its pharmacokinetics (PK). Analysis and understanding of the PK properties of CBD is critical to its future use as a therapeutic compound in a wide range of clinical settings, particularly regarding dosing regimens and routes of administration. Therefore, the aim of this systematic review was to collate and analyse all available CBD PK data recorded in humans and to highlight gaps in the literature.

## Methods

### Search strategy

The systematic review was carried out in accordance with PRISMA (Preferred Reporting Items for Systematic Reviews and Meta-Analyses) guidelines (Moher et al., 2009). A systematic search of PubMed and EMBASE (including MEDLINE) was conducted to retrieve all articles reporting pharmacokinetic data of CBD in humans. Search terms included: CBD, cannabidiol, Epidiolex, pharmacokinetics, C_max_, plasma concentrations, plasma levels, half-life, peak concentrations, absorption, bioavailability, AUC, T_max_, C_min_, and apparent volume of distribution. No restrictions were applied to type of study, publication year, or language. The searches were carried out by 14 March 2018 by two independent researchers.

### Eligibility criteria

The titles and abstracts of retrieved studies were examined by two independent researchers, and inappropriate articles were rejected. Inclusion criteria were as follows: an original, peer-reviewed paper that involved administration of CBD to humans, and included at least one pharmacokinetic measurement as listed in the search strategy.

### Data acquisition

The included articles were analyzed, and the following data extracted: sample size, gender, administration route of CBD, source of CBD, dose of CBD, and any pharmacokinetic details. Where available, plasma mean or median C_max_ (ng/mL) were plotted against CBD dose (mg). Similarly, mean or median T_max_ and range, and mean or median area under the curve (AUC_0−t_) and SD were plotted against CBD dose (mg). The source/supplier of the CBD was also recorded. No further statistical analysis was possible due to sparsity of data and heterogeneity of populations used. All studies were assessed for quality using an amended version of the National Institute for Health (NIH), National Heart, Lung and Blood Institute, Quality Assessment Tool for Before-After (Pre-Post) Studies with No Control Group (National Institute for Health, 2014). A sample size of ≤ 10 was considered poor, between 11 and 19 was considered fair, and ≥20 was considered good (Ogungbenro et al., 2006).

### Definitions of PK parameters

T_max_: Time to the maximum measured plasma concentration.

C_max_: Maximum measured plasma concentration over the time span specified.

t_1/2_: Final time taken for the plasma concentration to be reduced by half.

AUC_0−t_: The area under the plasma concentration vs. time curve, from time zero to “t.”

AUC_0−inf_: The area under the plasma concentration vs. time curve from zero to t calculated as AUC_0−t_ plus the extrapolated amount from time t to infinity.

K_el_: The first-order final elimination rate constant.

## Results

**Figure 1:**
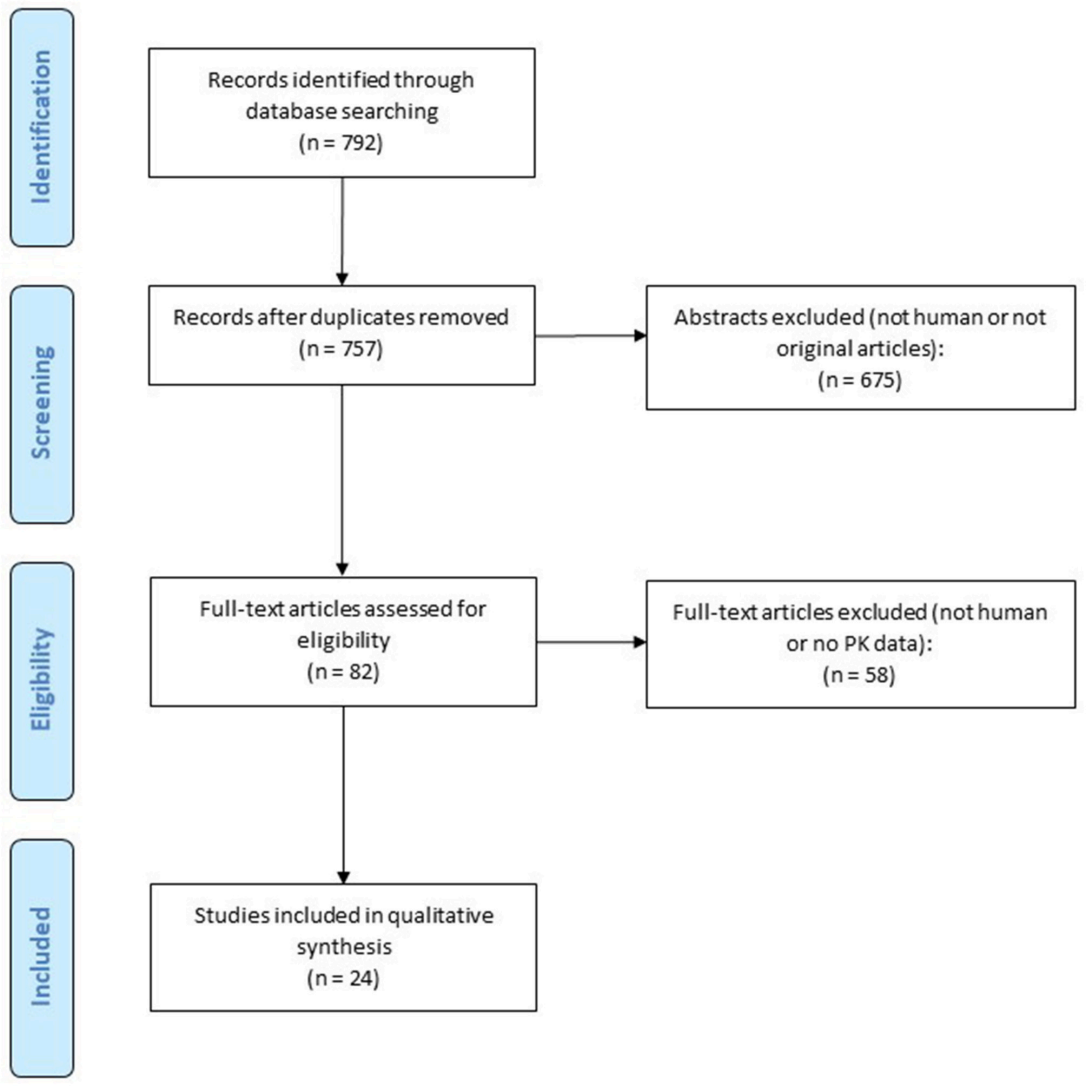
Flow chart for study retrieval and selection.

In total, 792 records were retrieved from the database searching, 24 of which met the eligibility criteria (Figure 1). Table 1 summarizes each included study. Routes of administration included intravenous (i.v.) (*n* = 1), oromucosal spray (*n* = 21), oral capsules (*n* = 13), oral drops (*n* = 2), oral solutions (*n* = 1), nebuliser (*n* = 1), aerosol (*n* = 1), vaporization (*n* = 1), and smoking (*n* = 8). CBD was administered on its own in 9 publications, and in combination with THC or within a cannabis extract in the remainder. One study was conducted in children with Dravet syndrome, while the remainder were conducted in healthy adult volunteers (Devinsky et al., 2018b). Overall, the included studies were of good quality (Supplementary Table 1). However, many studies had small sample sizes. Additionally, not all studies included both males and females, and frequent cannabis smokers were included in a number of studies. Thus, interpretation and extrapolation of these results should be done with caution.

**Table 1:** Human studies reporting pharmacokinetic (PK) parameters for cannabidiol (CBD).

**References**	**Total n, sex**	**Administration**	**Source**	**CBD dose**	**Plasma**^**a**^**PK details**							**Other**
					**T_max_ (median, range),^b^ hrs**	**C_max_ (mean, SD)^b^ ng/mL**	**AUC_0-t_ (mean, SD)^b^ h × ng/mL**	**AUC_0-inf_ (mean, SD) h × ng/mL**	**K_el_ (mean, SD) 1/h**	**t_1/2_ (mean, SD)^b^ h**	**CL/F (mean, SD) L/h**	**Mean (SD)**
Ohlsson et al., 1986	5, M, infrequent to frequent cannabis smokers	i.v.	In lab	20 mg		686 (239)	ng/ml min × 10^−3^ = 16.67 (3.23)			24 (6)	74.4 (14.4)	Distribution volume: 32.7 (8.6) l/kg.
**‘'**		Smoking	In lab	19.2 ± 0.3mg		110 (55)	ng/ml min × 10^−3^ = 4.85 (1.72)			31 (4)		Estimated systemic availability (%) from smoking: 31 (13)
Consroe et al., 1991	15, M/F	Oral capsules	NIDA	10 mg/kg/day daily for 6 weeks						2–5 days		
Guy and Robson, 2004b	12, M/F	Oromucosal spray sublingual (CBD and THC)	GW	10 mg	1.63 (SD 0.68)	2.5 (1.83)	6.81 (4.33)	7.12 (4.31)		1.44 (0.79)		
		Oromucosal spray buccal (CBD and THC)	GW	10 mg	2.79 (SD 1.31)	3.02 (3.15)	6.4 (4.62)	6.8 (4.46)		1.81 (2.05)		
		Oromucosal spray oro-pharyngeal (CBD and THC)	GW	10 mg	2.04 (SD 1.13)	2.61 (1.91)	7.81 (5.13)	8.28 (5.32)		1.76 (0.8)		
		CBME oral capsule (CBD and THC)	GW	10 mg	1.27 (SD 0.84)	2.47 (2.23)	5.76 (4.94)	6.03 (4.97)		1.09 (0.46)		
Guy and Robson, 2004a	24, M	Oromucosal spray sublingual (CBD and THC)	GW	10 mg	4.22	3.33	11.34	11.97		1.81		
Guy and Flint, 2004	6 M/F	Nebuliser (CBD and THC)	GW	20 mg	0.6 (0.08–1)	9.49 (8.01)	9.41 (10.8)	12.11 (10.83)	0.98 (0.58)	1.1 (0.97)		
		Aerosol (with THC)	GW	20 mg	2.35 (0.75–6)	2.6 (1.38)	5.43 (5.88)	13.53 (3.64)	0.43 (0.26)	2.4 (2.02)		
		Sublingual drops (CBD)	GW	20 mg	2.17 (1–4)	2.05 (0.92)	2.60 (3.45)					
		Sublingual drops (CBD and THC)	GW	20 mg	1.67 (1–3)	2.58 (0.68)	3.49 (2.65)	9.65 (4.02)	0.37 (0.114)	1.97 (0.62)		
Nadulski et al., 2005a	24, M/F	Oral capsule (CBD and THC)	Scherer GmbH & Co. KG, Eberbach,Germany	5.4mg once a week for 3 weeks	Mean 0.99 (0.5–2)	0.93 (range 0–2.6)	Mean 4.35, range (2.7–5.6)					
	12, M/F	Oral capsule (CBD and THC) and breakfast consumed 1 hour after	Scherer GmbH & Co. KG, Eberbach,Germany	5.4mg once a week for 3 weeks	Mean 1.07 (0.5–2)	1.13 (range 0.39–1.9)	Mean 4.4 (range 2.5–5.3)					
Nadulski et al., 2005b	24, M/F	Cannabis extract	Sigma	5.4 mg	Mean 1.0 (0.5–2.0)	0.95 (range 0.3–2.57)						
Karschner et al., 2011	9, M/F cannabis smokers	Oromucosal spray (Sativex: CBD and THC)	GW	5 mg	3.6 (1.0–5.5)	Mean (SE): 1.6 (0.4)	4.5 (SE 0.6)					
				15 mg	4.6 (1.2–5.6)	Mean (SE): 6.7 (2.0)	18.1 (SE 3.6)					
Schwope et al., 2011	10, M/F, usual infrequent cannabis smokers	Cannabis cigarette	NIDA	2 mg	0.25 (0.25–0.50 h) whole blood/plasma	Median (range): plasma 2 (< LOQ−3.4)						
Eichler et al., 2012	9, M	Oral capsules (CBD and THC)	Cannapharm AG	Heated CBD (27.8 mg CBD: 0.8 mg CBDA)	0.83 (SD 0.17)	pmol/mL: 0.94 (0.22)	pmol h/moL 3.68 (1.34)					
				Unheated 14.8 mg CBD:10.8 mg CBDA)	1.17 (SD 0.39)	3.95 (0.92) pmoL/mL	pmol h/mol 7.67 (2.06)					
Lee et al., 2012	10, M/F, cannabis smokers	Cannabis cigarette	NIDA	2 mg	Median 0.25 (oral fluid)	0.03 (oral fluid)						
Sellers et al., 2013	60, M/F	Oromucosal spray (CBD and THC)	GW	20 mg, 5 days	1.4 (0, 8.45)	1.5 (0.78)	6.1 (5.76)	14.8 (7.87)				
	51, M/F			90 mg – 60 mg, 5 days	1.5 (0–6.45)	4.8 (3.4)	38.9 (33.75)	60.3 (37.71)				
Stott et al., 2013b	12, M	Oromucosal spray (CBD and THC)	GW	10 mg (fed state)	4.00 (3.02–9.02);	3.66 (2.28)	23.13 (9.29)	20.21 (8.43)	0.155 (0.089)	5.49 (2.17)	533 (318)	
Stott et al., 2013a	24, M	Oromucosal spray (CBD and THC)	GW	5 mg single dose	Mean 1.00 (0.75–1.50)	0.39 (0.08)	0.82 (0.33)	1.66 (0.51)	0.173 (0.084)	5.28 (3.28)	3,252 (1,002)	
				10 mg single dose	Mean 1.39 (0.75–2.25)	1.15 (0.74)	4.53 (3.53)	5.64 (4.09)	0.148 (0.079)	6.39 (4.48)	2,546 (1,333)	
				20 mg single dose	Mean 1.00 (0.75–1.75)	2.17 (1.23)	9.94 (9.02)	13.28 (12.86)	0.123 (0.097)	9.36 (6.81)	3,783 (4,299)	
				5 mg, 9 days	Mean 1.64 (1.00–4.02)	0.49 (0.21)	2.52 (0.73)					
				10 mg, 9 days	Mean 1.27 (0.75–2.52)	1.14 (0.86)	6.66 (3.10)					
				20 mg 9 days	Mean 2.00 (1.02–6.00)	3.22 (1.90)	20.34 (7.29)					
Stott et al., 2013c	36, M	Oromucosal spray (CBD and THC)	GW	10 mg (3 groups)	1.00 (0.50–4.00); 1.38 (0.75–6.00); 1.15 (0.50–3.02)	1.03 (0.81); 0.66 (0.37); 0.63 (0.43)	3.23 (2.13); 1.82 (1.03); 1.83 (1.19)	5.10 (3.06); 3.54 (0.80); 3.00 (1.43)	0.148(0.108); 0.122 (0.111); 0.224 (0.158)	10.86(12.71); 7.81 (3.00); 5.22 (4.51)	2817 (1913); 2998 (896); 4,741 (3,835)	Varea/F (L): 28312 (19355); 31994 (12794); 26298 (14532)
				15 mg	4.5 (1.2–5.6)	Mean (SE): 6.7 (2.0)						
Newmeyer et al., 2014	24, M/F, frequent or occasional cannabis smokers	Cannabis cigarette (frequent smokers)	NIDA	2 ± 0.6 mg	0.5 (0.5–1)	Median (range): 14.8 (1.4–162)	Median (range): 29 (4.7–211)					
		Cannabis cigarette (occasional smokers)		2 ± 0.6 mg	1 (0.5–2)	Median (range): 7 (1.9–111)	Median (range): 11.6 (4.1–185)					
Desrosiers et al., 2014	21, M/F frequent and occasional smokers	Cannabis cigarette (frequent smokers)	NIDA	2 mg	0.5 (0.0–1.1)	1.1 (0.0–1.6)						
		Cannabis cigarette (occasional smokers)		2 mg	0 (0–500)	0 (0–1300)						
Manini et al., 2015	17, M/F	Oral capsules Co-administered with i.v. fentanyl	GW	400 mg	3 and 1.5 (plasma) and 6 and 2 (urine)	Plasma: 181.2 (39.8) and 114.2 (9.5); Urine: 4600 and 2900	704 (283) and 482 (314) mcg*hr/dL					
				800 mg	3 and 4 (plasma) and 4 and 6 (urine)	Plasma: 221 (35.6) and 157.1 (49.0); Urine: 3700 and 2800	867 (304) and 722 (443) mcg*hr/dL					
Haney et al., 2016	8, M/F cannabis smokers	Oral capsules	STI pharmaceuticals	800 mg	Mean 3 (2–6)	77.9 (range 1.6–271.9)						
Cherniakov et al., 2017a	9, M	Oral capsules with piperine pro-nanolipospeheres (CBD and THC)	STI pharmaceuticals	10 mg	1 (0.5–1.5)	2.1 (0.4)	6.9 (1.3)					
		Oromucosal spray (CBD and THC; Sativex®)		10 mg	3 (1–5)	0.5 (0.1)	3.1 (0.4)					
Swortwood et al., 2017	20, M/F Cannabis smokers	Cannabis cigarettes – frequent smokers	NIDA	1.5 mg	Mean 0.29 (0.17–1.5) (oral fluid)	93.3 (range 0.65–350) (oral fluid)						
		Cannabis cigarettes – occasional smokers	NIDA	1.5 mg	Mean 0.17 (oral fluid)	55.9 (range 2.5–291) (oral fluid)						
		Cannabis containing brownie – frequent smokers	NIDA	1.5 mg	Mean 0.53 (0.17–1.5) (oral fluid)	8.0 (range 0.48–26.3) (oral fluid)						
		Cannabis containing brownie – occasional smokers	NIDA	1.5 mg	Mean 0.47 (0.17–1.5) (oral fluid)	5.9 (range 2.1–11.4) (oral fluid)						
		Vaporization – frequent smokers	Volcano® Medic, Storz & Bickel, Tuttlingen, Germany	1.5 mg	Mean 0.29 (0.17–1.5) (oral fluid)	76.3 (range 2.3–339) (oral fluid)						
		Vaporization – occasional smokers	Volcano® Medic, Storz & Bickel, Tuttlingen, Germany	1.5 mg	Mean 0.17 (oral fluid)	28.2 (range 0.23–167) (oral fluid)						
Atsmon et al., 2017b	15, M	CBD extract >93% in a PTL101 formulation (oral gelatin matrix pellet technology)Sublingual/buccal	AiFame-AiLab GmbH (CBD), Gelpell AG (capsules)	10 mg	3.0 (2.0–4.0)	3.22 (1.28)	9.64 (3.99)	10.31 (4.14)		2.95 (2.58)		
				100 mg	3.5 (1.5–5.0)	47.44 (20.14)	149.54 (34.34)	153.04 (34.7)		3.59 (0.26)		
		Oromucosal spray (CBD and THC)	GW	10 mg	3.5 (1.0–5.0)	2.05 (1.1)	7.3 (2.86)	7.81 (2.81)	0.33 (0.09)	2.31 (0.72)		
Atsmon et al., 2017a	15, M	CBD and THC in a PTL401 capsule (self-emulsifying oral drug delivery system)	STI pharmaceuticals	10 mg	1.25 (0.5–4.0)	2.94 (0.73)	9.85 (4.47)	10.52 (4.53)	0.29 (0.17)	3.21 (1.62)		
Devinsky et al., 2018b	34, children	Oral solution	GW	2.5 mg			70.23 (mean from 3 groups)					
				5 mg/kg/day			241					
				10 mg/kg/day			722					
				20 mg/kg/day			963					

a, b *Unless otherwise stated. PK, pharmacokinetics; CBD, cannabidiol; THC, Tetrahydrocannabinol; M, male; F, female; AUC, area under the curve; Conc., concentration; GW, GW pharmaceuticals; NIDA, US national institute on drug abuse; LOQ, limit of quantification; IV, intravenous; CBME, cannabis based medicine extract; Min(s), min(s)*.

### C_max_, T_max_, and area under the curve

**Figure 2:**
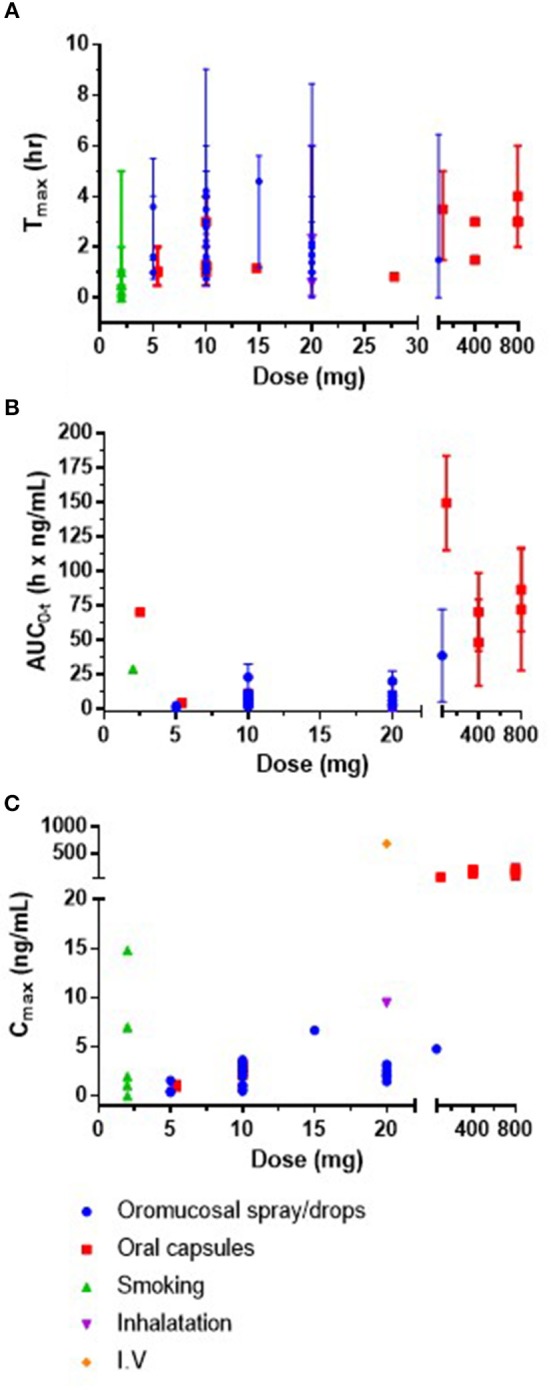
**(A)** Mean or median Tmax (h) and range against CBD dose (mg) **(B)** mean or median area under the curve (AUC0-t) (h × ng/mL) and SD against CBD dose (mg) and **(C)** plasma mean or median concentration max (Cmax; ng/mL) against CBD dose (mg). It was not possible to present error bars for Cmax as SD and SEM were both reported in the data. IV, intravenous; SD, standard deviation; SEM, standard error of the mean.

Within the 25 included studies, C_max_ was reported on 58 occasions (for example within different volunteer groups or doses in a single study), T_max_ on 56 occasions and area under the curve (AUC_0−t_) on 45 occasions. These data from plasma/blood are presented in Figures 2A–C. The AUC_0−t_ and C_max_ of CBD is dose-dependent, and T_max_ occurs between 0 and 5 h, but does not appear to be dose-dependent.

#### Oromucosal drops/spray

A number of trials in humans were conducted by Guy and colleagues to explore administration route efficiency of sprays, an aerosol, and a nebuliser containing CBD or CBD and THC (CBD dose 10 or 20 mg) (Guy and Flint, 2004; Guy and Robson, 2004a,b). Oromucosal spray, either buccal, sublingual, or oropharyngeal administration, resulted in mean C_max_ between 2.5 and 3.3 ng/mL and mean T_max_ between 1.64 and 4.2 h. Sublingual drops resulted in similar C_max_ of 2.05 and 2.58 ng/mL and T_max_ of 2.17 and 1.67 h, respectively. Other oromucosal single dose studies reported C_max_ and T_max_ values within similar ranges (Karschner et al., 2011; Atsmon et al., 2017b).

Minimal evidence of plasma accumulation has been reported by chronic dosing studies over 5–9 days (Sellers et al., 2013; Stott et al., 2013a). C_max_ appears to be dose-dependent. A dose of 20 mg/day resulted in a mean C_max_ of 1.5 ng/mL and mean AUC_0−t_ of 6.1 h × ng/mL while 60 mg/day equated to a mean C_max_ of 4.8 ng/mL and AUC_0−t_ was 38.9 h × ng/mL (Sellers et al., 2013). In another study, C_max_ increased dose-dependently from 0.4 to 1.2 and 2.2 ng/mL following 5, 10, and 20 mg single doses, respectively, and from 0.5 to 1.1 and 3.2 ng/mL, respectively following chronic dosing over 9 consecutive days (Stott et al., 2013a). There was a significant increase in time-dependent exposure during the chronic treatment. Mean AUC_0−t_ for the single doses were 0.8, 4.5, 9.9, and 2.5, 6.7, and 20.3 for the chronic dosing schedule, respectively. T_max_ does not appear to be dose-dependent, nor affected by acute or chronic dosing schedules.

Stott et al. reported an increase in CBD bioavailability under fed vs. fasted states in 12 men after a single 10 mg dose of CBD administered through an oromucosal spray which also contained THC (Stott et al., 2013a,b). Mean AUC and C_max_ were 5- and 3-fold higher during fed conditions compared to fasted (AUC_0−t_ 23.1 vs. 4.5; C_max_ 3.7 vs. 1.2 ng/mL). T_max_ was also delayed under the fed state (4.0 vs. 1.4 h).

In children, Devinsky et al. reported mean AUC as 70, 241, 722, and 963 h × ng/mL in groups receiving 2.5, 5, 10, and 20 mg/Kg/day of CBD in oral solution (Devinsky et al., 2018b).

#### Oral intake

C_max_ and AUC following oral administration also appears to be dose dependent. A dose of 10 mg CBD resulted in mean C_max_ of 2.47 ng/mL at 1.27 h, and a dose of 400 or 800 mg co-administered with i.v. fentanyl (a highly potent opioid) to examine its safety resulted in a mean C_max_ of 181 ng/mL (at 3.0 h) and 114 ng/mL (at 1.5 h) for 400 mg, and 221 ng/mL (at 3.0 h) and 157 ng/mL (at 4.0 h) for 800 mg, in 2 sessions, respectively (Guy and Robson, 2004b; Manini et al., 2015). A dose of 800 mg oral CBD in a study involving 8 male and female cannabis smokers, reported a mean C_max_ of 77.9 ng/mL and mean T_max_ of 3.0 h (Haney et al., 2016). Although, an increase in dose corresponds with an increase in C_max_, the C_max_ between the higher doses of CBD does not greatly differ, suggesting a saturation effect (e.g., between 400 and 800 mg).

One hour after oral capsule administration containing 5.4 mg CBD in males and females, mean C_max_ was reported as 0.93 ng/mL (higher for female participants than male) (Nadulski et al., 2005a). A subset (*n* = 12) consumed a standard breakfast meal 1 h after the capsules, which slightly increased mean C_max_ to 1.13 ng/mL. CBD remained detectable for 3–4 h after administration (Nadulski et al., 2005b).

Cherniakov et al. examined the pharmacokinetic differences between an oromucosal spray and an oral capsule with piperine pro-nanolipospheres (PNL) (both 10 mg CBD) in 9 men. The piperine-PNL oral formulation had a 4-fold increase in C_max_ (2.1 ng/mL vs. 0.5 ng/mL), and a 2.2-fold increase in AUC_0−t_ (6.9 vs. 3.1 h × ng/mL), while T_max_ was decreased (1.0 vs. 3.0 h) compared to the oromucosal spray (Cherniakov et al., 2017a). This group further developed self-emulsifying formulations and reported again an increased bioavailability and increased C_max_ within a shorter time compared to a reference spray (Atsmon et al., 2017a,b).

#### Intravenous administration

The highest plasma concentrations of CBD were reported by Ohlsson et al. following i.v. administration of 20 mg of deuterium-labeled CBD. Mean plasma CBD concentrations were reported at 686 ng/mL (3 min post-administration), which dropped to 48 ng/mL at 1 h.

#### Controlled smoking and inhalation

After smoking a cigarette containing 19.2 mg of deuterium-labeled CBD, highest plasma concentrations were reported as 110 ng/mL, 3 min post dose, which dropped to 10.2 ng/ml 1 h later (Ohlsson et al., 1986). Average bioavailability by the smoked route was 31% (Ohlsson et al., 1986). A nebuliser resulted in a C_max_ of 9.49 ng/mL which occurred at 0.6 h, whereas aerosol administration produced C_max_ (2.6 ng/mL) at 2.35 h (Guy and Flint, 2004). In 10 male and female usual, infrequent cannabis smokers, C_max_ was 2.0 ng/mL at 0.25 h after smoking a cigarette containing 2 mg of CBD (Schwope et al., 2011). CBD was detected in 60% of whole blood samples and in 80% of plasma samples at observed C_max_, and no longer detected after 1.0 h. A study in 14 male and female cannabis smokers reported 15.4% detection in frequent smokers with no CBD detected in occasional smokers in whole blood analysis (Desrosiers et al., 2014). In plasma however, there was a 53.8 and 9.1% detection in the frequent and occasional groups, with corresponding C_max_ of 1.1 ng/mL in the frequent group, and below limits of detection in the occasional group.

### Half-life

The mean half-life (t_1/2_) of CBD was reported as 1.1 and 2.4 h following nebuliser and aerosol administration (20 mg) (Guy and Flint, 2004), 1.09 and 1.97 h following single oral administration (10 and 20 mg) (Guy and Flint, 2004; Guy and Robson, 2004b), 2.95 and 3.21 h following 10 mg oral lipid capsules (Atsmon et al., 2017a,b), between 1.44 and 10.86 h after oromucosal spray administration (5–20 mg) (Guy and Robson, 2004b; Sellers et al., 2013; Stott et al., 2013a,b; Atsmon et al., 2017b), 24 h after i.v. infusion, 31 h after smoking (Ohlsson et al., 1986), and 2–5 days after chronic oral administration (Consroe et al., 1991).

### Elimination rate

Mean elimination rate constant (K_el_ [1/h]) has been reported as 0.148 in fasted state, and 0.155 in fed state after 10 mg CBD was administered in an oromucosal spray also containing THC (Stott et al., 2013a,b). After single doses of 5 and 20 mg CBD, mean K_el_ (1/h) was reported as 0.173 and 0.123 (Stott et al., 2013a). Following 20 mg CBD administration through a nebuliser and pressurized aerosol, mean K_el_ was reported as 0.98 and 0.43, respectively, while 20 mg CBD administered as sublingual drops was reported as 0.37 (Guy and Flint, 2004).

### Plasma clearance

Plasma apparent clearance, CL/F (L/h) has been reported to range from 2,546 to 4,741 in a fasted stated following 10 mg CBD administered via oromucosal spray (Stott et al., 2013a,c). This value decreases to 533 following the same concentration in a fed state (Stott et al., 2013b). A plasma apparent clearance of 3,252 and 3,783 was reported following 5 and 20 mg single doses of CBD via oromucosal spray (Stott et al., 2013a). Ohlsson et al. reported plasma apparent clearance as 74.4 L/h following i.v. injection (Ohlsson et al., 1986).

### Volume of distribution

Mean apparent volume of distribution (V/F [L]) was reported as 2,520 L following i.v. administration (Ohlsson et al., 1986). Following single acute doses through oromucosal spray administration, apparent volume of distribution was reported as 26,298, 31,994, and 28,312 L (Stott et al., 2013a).

## Discussion

The aim of this study was to review and analyse all available PK data on CBD in humans. Only 8 publications reported PK parameters after administering CBD on its own, and the others were in combination with THC/cannabis. Only 1 study reported the bioavailability of CBD in humans (31% following smoking). From the analysis of these papers, the following observations were made; peak plasma concentrations and area under the curve (AUC) are dose-dependent and show minimal accumulation; C_max_ is increased and reached faster following i.v., smoking or inhalation; C_max_ is increased and reached faster after oral administration in a fed state or in a pro-nanoliposphere formulation; T_max_ does not appear to be dose-dependent; and half-life depends on dose and route of administration. Overall, considerable variation was observed between studies, although they were very heterogeneous, and further work is warranted.

Human studies administering CBD showed that the AUC_0−t_ and C_max_ are dose-dependent, and T_max_ mostly occurred between 1 and 4 h. Animal studies in piglets, mice, and rats also all demonstrate a dose-dependent relationship between CBD and both plasma and brain concentrations (Long et al., 2012; Hammell et al., 2016; Garberg et al., 2017), suggesting that human brain concentrations will also be dose-dependent. Ten publications in this review reported the half-life of CBD which ranged from 1 h to 5 days and varies depending on the dose and route of administration. Very limited data was available for detailed analysis on the elimination rate, apparent clearance or distribution of CBD in humans.

Plasma levels of CBD were increased when CBD was administered with food or in a fed state, or when a meal is consumed post-administration. Oral capsules with piperine pro-nanolipospheres also increased AUC and C_max_. This is also demonstrated in animal studies; co-administration of lipids with oral CBD increased systemic availability by almost 3-fold in rats (Zgair et al., 2016) and a pro-nanoliposphere formulation increased oral bioavailability by about 6-fold (Cherniakov et al., 2017b). As CBD is a highly lipophilic molecule, it is logical that CBD may dissolve in the fat content of food, increasing its solubility, and absorption and therefore bioavailability as demonstrated by numerous pharmacological drugs (Winter et al., 2013). Thus, it may be advisable to administer CBD orally in a fed state to allow for optimal absorption.

Only one study used intravenous administration of CBD and reported PK details, which could be a beneficial route of administration in some acute indications. Results from other routes such as rectal, transdermal, or intraperitoneal have also not been published in humans, although transdermal CBD gel and topical creams have been demonstrated to be successful in animal studies (Giacoppo et al., 2015; Hammell et al., 2016). Interestingly, intraperitoneal (i.p.) injection of CBD corresponded to higher plasma and brain concentrations than oral administration in mice, however in rats, similar concentrations were observed for both administration routes, and brain concentrations were in fact higher following oral compared to i.p. route (Deiana et al., 2012). No published data exists on the tissue distribution of CBD in humans. Although plasma levels of CBD do not show accumulation with repeated dosing, it is possible that there may be tissue accumulation.

Only one study in this review was conducted in children (*n* = 34) (Devinsky et al., 2018b). Children (4–10 years) with Dravet syndrome were administered an oral solution of CBD and AUC was reported to increase dose-dependently. It is important to emphasize the statement that children are not small adults, and there are many differences in their pharmacokinetic and pharmacodynamic profiles. Absorption, excretion, metabolism, and plasma protein binding are generally reduced in children compared to adults, and apparent volume of distribution is generally increased (Fernandez et al., 2011). These parameters need to be explored fully for CBD in order to understand and advise dose adjustments.

Within the adult studies, inter- and intra-subject variability was observed in studies, and it remains to be seen whether i.v. and other routes of administration that by-pass initial metabolism will alleviate this issue. Interestingly, although each of the subject's weight was taken into account, none of the studies addressed subject fat content as a factor in their exclusion criteria; as muscle can weigh more than the same proportion of fat. It is well-known that cannabinoids are highly lipophilic compounds and accumulate in fatty tissue which can then be released gradually (Gunasekaran et al., 2009). It may be of benefit in future study to either put in place more stringent exclusion criteria and measure subject fat content or assess the possible accumulation of CBD in fatty tissue. Differences in metabolism, distribution and accumulation in fat, and in biliary and renal elimination may be responsible for prolonged elimination half-life and variable pharmacokinetic outcomes. CBD use is widespread and has been recommended for use by the FDA in childhood-onset epilepsy. CBD also displays therapeutic promise in other disorders such as schizophrenia and post-traumatic stress disorder. If we are to understand the actions of CBD in those disorders and increase the success rate for treatment, these groups of patients and their distinct characteristics must be assessed as they may not be comparable to a healthy volunteer population.

A systematic review in 2014 concluded that CBD generally has a low risk of clinically significant drug-interactions (Stout and Cimino, 2014). A few studies in the current review included examination of drug-drug interactions with CBD. GW Pharmaceuticals performed a clinical trial investigating the pharmacokinetic interaction between CBD/THC spray (sativex) and rifampicin (cytochrome P450 inducer), ketoconazole, and omeprazole (cytochrome P450 inhibitors) (Stott et al., 2013c). Authors concluded overall that CBD in combination with the drugs were well-tolerated, but consideration should be noted when co-administering with other drugs using the CYP3A4 pathway. Caution is also advised with concomitant use of CBD and substrates of UDP-glucuronosyltransferases UGT1A9 and UGT2B7, and other drugs metabolized by the CYP2C19 enzyme (Al Saabi et al., 2013; Jiang et al., 2013). Manini et al. co-administered CBD with i.v. fentanyl (a high potency opioid) which was reported as safe and well-tolerated (Manini et al., 2015). In a number of trials with CBD in children with severe epilepsy, clobazam concentrations increased when CBD was co-administered and dosage of clobazam had to be reduced in some patients in one study (Geffrey et al., 2015; Devinsky et al., 2018b). Gaston and colleagues performed a safety study in adults and children in which CBD was administered with commonly-used anti-epileptic drugs (AEDs) (Gaston et al., 2017). Most changes in AED concentrations were within acceptable ranges but abnormal liver function tests were reported in those taking valproate and authors emphasized the importance of continued monitoring of AED concentrations and liver function during treatment with CBD.

Limitations of this review should be acknowledged. Different population types including healthy and patient populations and cannabis naïve or not were all grouped together which may impede generalizability. The proportions of men and women in each study were also not uniform, and it is still being elucidated whether men and women have distinct pharmacokinetic profiles with regards to cannabinoids (Fattore and Fratta, 2010). One study suggested that the PK of CBD was different in their female volunteers (Nadulski et al., 2005a). It should also be mentioned that CBD is currently not an approved product with a pharmacopeia entry so using different sources of CBD that are subject to different polymeric forms, different particle sizes, and different purities may also affect the PK profiles observed. It is important for future work that researchers record the source of the CBD material used so that results have the highest chance of being replicated. Despite a thorough search of the two databases chosen, the addition of more databases may have widened the search to increase the number of results and hence improve the reliability and validity of the findings. However, the review was carried out by two independent reviewers, and searches generated were analyzed separately and then compared.

In conclusion, this review demonstrates the lack of research in this area, particularly in routes of administration other than oral. An absence of studies has led to failure in addressing the bioavailability of CBD despite intravenous formulations being available. This is of critical importance due to the popularity of CBD products and will help interpret other PK values. Standardized and robust formulations of CBD and their PK data are required for both genders, with consideration of other factors such as adiposity, genetic factors that might influence absorption and metabolism, and the effects of disease states.

## Author contributions

SM, SO, and AY: substantial contributions to the conception or design of the work. SM: writing of the manuscript. SM and NS: database searching and data extraction. All authors: the analysis and interpretation of data for the work; drafting the work or revising it critically for important intellectual content; final approval of the version to be published; agreement to be accountable for all aspects of the work in ensuring that questions related to the accuracy or integrity of any part of the work are appropriately investigated and resolved.

### Conflict of interest statement

AY was employed by company Artelo Biosciences. The remaining authors declare that the research was conducted in the absence of any commercial or financial relationships that could be construed as a potential conflict of interest.
