# Supplementary material for: A Systematic Review on the Pharmacokinetics of Cannabidiol in Humans
Source: Front Pharmacol. 2018 Nov 26;9:1365. doi: 10.3389/fphar.2018.01365 (PMC6275223; doi:10.3389/fphar.2018.01365)
Supplement: Supplementary file 1 [file Data_Sheet_1.PDF]

Supplementary Table 1. Quality assessment of publications reporting pharmacokinetic parameters of cannabidiol in humans. Adapted from the NIH National Heart, Lung and Blood Institute, Quality Assessment Tool for Before-After (Pre-Post) Studies with No Control Group.

Study	Q1	Q2	Q3	Q4	Q5	Q6	Overall
Ohlsson, 1986[32]	Good	Poor	Fair	Poor	Good	Good	Fair
Consroe, 1991[35]	Good	Poor	Poor	Fair	Good	Good	Fair
Guy, 2004[20]	Good	Good	Good	Poor	Good	Good	Good
Guy,2004[18]	Good	Good	Fair	Good	Good	Good	Good
Guy, 2004[19]	Good	Good	Good	Fair	Good	Good	Good
Nadulski, 2005[28]	Good	Fair	Good	Good	Good	Good	Good
Nadulski, 2005[29]	Good	Poor	Good	Good	Good	Good	Good
Karschner, 2011[21]	Good	Good	Fair	Poor	Good	Good	Good
Schwope, 2011[33]	Good	Good	Fair	Poor	Fair	Good	Good
Eichler, 2012[52]	Good	Good	Fair	Poor	Good	Good	Good
Lee, 2012[53]	Good	Good	Fair	Poor	Fair	Good	Good
Sellers, 2013[24]	Good	Good	Good	Good	Good	Good	Good
Stott, 2013[36]	Good	Good	Fair	Good	Good	Good	Good
Stott, 2013[25]	Good	Good	Fair	Fair	Good	Good	Good
Stott, 2013[23]	Good	Good	Fair	Good	Good	Good	Good
Newmeyer, 2014[54]	Good	Good	Fair	Good	Fair	Good	Good
Desrosiers, 2014[34]	Good	Good	Fair	Good	Good	Good	Good
Manini, 2015[26]	Good	Good	Good	Fair	Good	Good	Good
Haney, 2016[27]	Good	Good	Good	Poor	Good	Good	Good
Cherniakov, 2017[30]	Good	Good	Fair	Poor	Good	Good	Good
Swortwood, 2017[55]	Good	Good	Fair	Good	Good	Good	Good
Atsmon, 2017[31]	Good	Good	Fair	Fair	Good	Good	Good
Atsmon 2017[22]	Good	Good	Fair	Fair	Good	Good	Good
Devinsky, 2018[17]	Good	Fair	Good	Good	Fair	Good	Good

Q1: Was the study question or objective clearly stated?; **Q2:** Were eligibility/selection criteria for the study population pre-specified and clearly described?; **Q3:** Were the participants in the study representative of the general population?; **Q4:** Was the sample size sufficiently large to provide confidence in the findings?[1]; **Q5:** Was the test/service/intervention (i.e. cannabidiol administration) clearly described and delivered consistently across the study population?; **Q6:** Were the outcome measures (i.e. pharmacokinetic parameters) pre-specified, clearly defined, valid, reliable, and assessed consistently across all study participants?

[1]A sample size of ≤10 was considered poor, between 11 and 19 was considered fair, and ≥20 was considered good, based on Ogungbenro *et al.* (2006), J Biopharm Stat.
